# Role of Radiomics Features and Machine Learning for the Histological Classification of Stage I and Stage II NSCLC at [^18^F]FDG PET/CT: A Comparison between Two PET/CT Scanners

**DOI:** 10.3390/jcm12010255

**Published:** 2022-12-29

**Authors:** Francesco Dondi, Roberto Gatta, Domenico Albano, Pietro Bellini, Luca Camoni, Giorgio Treglia, Francesco Bertagna

**Affiliations:** 1 Nuclear Medicine, ASST Spedali Civili Brescia, 25123 Brescia, Italy; 2 Dipartimento di Scienze Cliniche e Sperimentali, Università degli Studi di Brescia, 25123 Brescia, Italy; 3 Nuclear Medicine, Università degli Studi di Brescia and ASST Spedali Civili Brescia, 25123 Brescia, Italy; 4 Nuclear Medicine, Imaging Institute of Southern Switzerland, Ente Ospedaliero Cantonale, 6500 Bellinzona, Switzerland; 5 Department of Nuclear Medicine and Molecular Imaging, Lausanne University Hospital, University of Lausanne, 1011 Lausanne, Switzerland; 6 Faculty of Biomedical Sciences, Università della Svizzera Italiana, 6900 Lugano, Switzerland

**Keywords:** PET/CT, radiomics, texture analysis, machine learning, lung cancer, FDG

## Abstract

The aim of this study was to compare two different PET/CT tomographs for the evaluation of the role of radiomics features (RaF) and machine learning (ML) in the prediction of the histological classification of stage I and II non-small-cell lung cancer (NSCLC) at baseline [^18^F]FDG PET/CT. A total of 227 patients were retrospectively included and, after volumetric segmentation, RaF were extracted. All of the features were tested for significant differences between the two scanners and considering both the scanners together, and their performances in predicting the histology of NSCLC were analyzed by testing of different ML approaches: Logistic Regressor (LR), k-Nearest Neighbors (kNN), Decision Tree (DT) and Random Forest (RF). In general, the models with best performances for all the scanners were kNN and LR and moreover the kNN model had better performances compared to the other. The impact of the PET/CT scanner used for the acquisition of the scans on the performances of RaF was evident: mean area under the curve (AUC) values for scanner 2 were lower compared to scanner 1 and both the scanner considered together. In conclusion, our study enabled the selection of some [^18^F]FDG PET/CT RaF and ML models that are able to predict with good performances the histological subtype of NSCLC. Furthermore, the type of PET/CT scanner may influence these performances.

## 1. Introduction

Non-small-cell lung cancer (NSCLC) is a frequent form of neoplasm with globally rising incidence, accounting for most cancer-related deaths worldwide [1,2,3]. The risk factors associated with the development of disease are mainly environmental, with cigarette smoking as the most important [4,5]. The three main histological types of NSCLC, according to World Health Organization (WHO), are adenocarcinoma (ADK), squamous cell carcinoma (SCC) and large cell carcinoma [4].

The correct diagnosis of NSCLC is often made in advanced stages, since the disease can become symptomatic only in these stages. In this setting, the main symptoms are presented by cough, hemoptysis, chest pain and dyspnea [4,6].

The correct diagnostic and staging assessment of NSCLC is performed with imaging evaluation with chest X-ray (XR) and chest computed tomography (CT) that are pivotal for the evaluation of disease. In this setting, such imaging modalities directly drive the treatment of the disease and are also able to perform distant staging, being useful even for its follow-up [4]. The clinical outcome of NSCLC is directly related to its stage at diagnosis: early stages are usually managed with surgical resection and good prognosis, while advanced and metastatic disease can benefit from adjuvant therapy [7,8,9,10,11].

In the recent years, positron emission tomography/computed tomography (PET/CT) with different tracers is emerging as a fundamental imaging modality for the assessment of a high amount of neoplastic and infectious diseases [12,13]. In this scenario, ^18^F-fluorodeoxyglucose ([^18^F]FDG) PET/CT is routinely performed for the staging of NSCLC and the usefulness of some semiquantitative parameters in predicting the prognosis of patients has been proved [14,15]. In this setting, the possible role of [^18^F]FDG PET/CT radiomics features (RaF) to differentiate between malignant and benign lesions in various organs has recently emerged and pulmonary nodules do not make exception [16,17]. Furthermore, it has also been reported that texture analysis is somehow able to differentiate between ADK and SCC, however with heterogeneous findings in terms of PET/CT acquisition, RaF extraction and results [18,19,20,21,22,23,24,25]. The populations considered in such studies were characterized by high heterogeneity in terms of clinicopathological features and, as mentioned, prognosis of patients is related to the stage of disease at the time of diagnosis. Furthermore, it is known that different scanners and protocols used for the acquisition and reconstruction of PET images are able to influence the extraction of RaF and therefore affect the results of such analysis [16,17,18].

The aim of this study is therefore to analyze the value of baseline [^18^F]FDG PET/CT RaF and ML models for the prediction of final histological diagnosis in patients with stage I and stage II NSCLC and also to assess the influence of different scanners on this scenario.

## 2. Materials and Methods

### 2.1. Patient Selection

We retrospectively screened our database in order to find patients submitted to our center to perform [^18^F]FDG PET/CT for the initial staging of NSCLC. The screening was performed from January 2014 until February 2022 and a total of 2332 subjects were selected. Inclusion criteria were the presence of a histologically proven diagnosis of stage I or stage II NSCLC, the presence of a baseline [^18^F]FDG PET/CT performed before any treatment and the presence of tracer uptake by NSCLC higher than liver uptake. After applying such inclusion criteria, 233 patients were included in the study.

Clinicopathological information including gender, age, size of NSCLC measured on histological evaluation, grading, lobe involved by the disease, therapy performed, TNM category and American Joint Commission on Cancer (AJCC) VIII Edition stage were collected. Furthermore, histological classification was collected and since only 6 patients had the presence of adenosquamous carcinoma, they were excluded from the present study. A total of 227 patients were therefore finally included in the study.

### 2.2. [^18^F]FDG PET/CT Acquisition and Interpretation

Patients fasted for at least 6 h before tracer injection and had a glucose blood level below 150 mg/dL (mean: 116, standard deviation [SD]: 19, range: 83–148). In order to perform PET/CT scan, 3.5–4.5 MBq/kg of [^18^F]FDG were intravenously injected to the patients and before images acquisition they were instructed to void. No contrast agent, intestinal preparation with purge or enteric contrast were used.

Images were acquired 60 min after radiotracer injection, from the vertex to the midthigh on two different PET/CT tomographs. The first one (scanner 1) was a Discovery 690 PET/CT (General Electric Company, Milwaukee, WI, USA) while the second (scanner 2) was a Discovery STE PET/CT (General Electric Company, Milwaukee, WI, USA). On both, standard acquisition parameters (CT: 80 mA, 120 kV without contrast; 2.5–4 min per bed PET-step, axial width 15 cm) and standard reconstruction parameters were used (256 × 256 matrix and 60 cm field of view). Furthermore, scanner 1 had LYSO (cerium-doped lutetium yttrium oxyorthosilicate) scintillator crystals with a decay time of 45 ns, while scanner 2 had BGO (bismuth germanate) scintillator crystals with a decay time of 300 ns. Scanners were not harmonized with a cross-calibration program and all PET/CT scans were acquired at free-breath, instructing the patients to have regular breathing. For anatomical correlation and to perform attenuation correction, a low dose CT at free breathing and without contrast agent was acquired for both the scanners. More in detail, CT acquisition parameters for scanner 1 were: 120 kV, fixed tube current ≈ 60 mAs (40–100 mAs), 64 slices × 3.75 mm and 3.27 mm interval, pitch 0.984:1, tube rotation 0.5 s. CT acquisition parameters for scanner 2 were: 120 kV, fixed tube current ≈ 73 mAs (40–160 mAs), 4 slices × 3.75 mm and 3.27 mm interval, pitch 1.5:1, tube rotation 0.8 s. Furthermore, on scanner 1 time of flight (TOF) and point spread function (PSF) algorithm were used for the reconstruction of images, with filter cut-off 5 mm, 18 subsets and 3 iterations. Moreover, on scanner 2 an ordered subset expectation maximization (OSEM) algorithm with filter cut-off 5 mm, 21 subsets and 2 iterations was used.

PET images were visually and semiquantitatively analyzed by a nuclear physician with at least 10 years of experience and every focal tracer uptake deviating from physiological distribution and background was regarded as suggestive of disease localization.

### 2.3. Radiomics Features Extraction

Before RaF extraction, PET/CT scan were segmented with LIFEx 2.20 software (http://www.lifexsoft.org, accessed on 10 September 2021) by manually drawing volume of interest (VOI) on hypermetabolic lesions [26]. RaF were extracted using Moddicom [27], a software library compliant with the Image Biomarker Standardization Initiative [28]. For each image series, a set of 216 features were extracted, grouped in three main families: morphological (12), grey-level histogram based (21) and textural (190).

### 2.4. Statistical Analysis

Statistical analyses were performed using R (version 3.6.3). The descriptive analysis of categorical variables comprised the calculation of simple and relative frequencies. The numeric variables were described as mean, SD, minimum and maximum (range).

The general statistical analysis line of the study was structured of various steps and was aimed at training a predictive model testing different Machine Learning (ML) approaches: Logistic Regressor (LR), k-nearest neighbors (kNN), Decision Tree (DT) and Random Forest (RF). Due to the nature of the used ML techniques, different approaches were used to cope with the feature selection strategy. For LR and kNN, for example, to reduce the complexity of the space, we used a Wilcoxon analysis after a 50-cross fold validation for all RaF and we removed the feature poorly correlated with the outcome. The 50-cross fold validation was performed randomly splitting the cohort in 80% for training and 20% per validation, 50 times. In this setting, a *p*-value ≤ 0.001 was considered as cutoff. DT and RF, on the other hand, did not need any preliminary feature selection strategy because they operate a feature selection exploiting a measure of the GINI index to split the node in the different branches (and this operation can be performed also with a relatively high number of variables). The ML models were than trained to find the best model and the corresponding most representative RaF. The different models were trained in the following ways:

- LR: a bivariate Logistic Regressor was trained using the RaF survived at the feature selection strategy. All the possible couple of RaF with a Spearman’s correlation coefficient lower than 0.3 were tested and only the LR models were both the *p*-values were lower than 0.05 were considered for the testing. This bivariate analysis was conducted in order to classify these couples based on the area under the curve (AUC) value of the receiver operating characteristic (ROC) analysis. The entire process was repeated in a 50 cross-fold validation, in order to be able to measure the mean and the SD of the AUCs, for each tested couple of RaF.
- kNN: kNN was trained with a 50 cross-fold validation technique for each couple of RaF tested for LR. This was done to assess the different performances between LR and kNN on the same couple of RaF. Again, mean and SD of the AUCs were measured.
- DT and RF were tested with a 50 cross-fold validation technique on all the available RaF. In this case, for each run of the cross-fold validation, only two model were trained (one for DT and one for RF) and the mean and the SD of the AUC were measured on the base of the 50 different training runs.

The entire aforementioned analyses were performed by considering the performances of scanner 1 alone, scanner 2 alone and both the scanners together.

## 3. Results

Among the total number of 227 patients included in the study, 147 were men (64.8%) and the mean age was 70 years (SD: 8, range: 38–87). The mean size of neoplasms was 32 mm (SD: 15, range: 7–69). Regarding histological classification of NSCLC, 169 patients (74.4%) had ADK while 58 (25.6%) were affected by SCC. In this setting, the lobe involved by disease was represented by left superior lobe (LSL) in 54 patients (23.8%), left inferior lobe (LIL) in 39 (17.2%), right superior lobe (RSL) in 82 (36.1%), middle lobe (ML) in 7 (3.1%) and right inferior lobe (RIL) in 47 (19.8%). For what concerns the TNM classification, 75 patients (33.0%) had a T1 disease, 114 (50.2%) had a T2 disease, while 38 (16.7%) had a T3 disease. In particular, 1 (0.4%) had a T1mi disease, 26 (11.5%) had a T1a disease, 31 (13.7%) had a T1b disease, 11 (4.8%) had a T1c disease, 2 (0.9%) had a T1aN1 disease, 3 (1.3%) had a T1bN1 disease, 1 (0.4%) had a T1cN1 disease, 59 (26.0%) had a T2a disease, 23 (10.1%) had a T2b disease, 23 (10.1%) had a T2aN1 disease, 9 (4.0%) had a T2bN1 disease and 38 (16.7%) had a T3 disease. As a consequence, the presence of nodal localization of disease was reported in 38 patients (16.7%) (Table 1).

According to the VIII edition of AJCC staging system, 128 patients (56.4%) had stage I disease while 99 (43.6%) had stage II disease. In particular, 27 (11.9%) subjects had stage IA1 disease, 31 (13.7%) had stage IA2 disease, 11 (4.8%) had stage IA3 disease, 59 (26.0%) had stage IB disease, 23 (10.1%) had stage IIA disease while 76 (33.5%) had stage IIB disease.

Data about the grading of disease were available only for 100 patients and in this setting 1 patient (1.0%) had a G1 disease, 42 (42.0%) had G2 disease, while 57 (57.0%) had G3 disease. Furthermore, a total of 142 (62.6%) scans were performed on the Discovery 690 tomograph (scanner 1), while 85 (37.4%) of them were acquired on the Discovery STE tomograph (scanner 2). Analyzing PET/CT acquisition depending on the tomograph used for their execution, in 107 (75.4%) scans performed on scanner 1 the presence of ADK was revealed while in 35 (24.6%) the presence of SCC was reported. On scanner 2, ADK was present in 66 (77.6%) subjects while SCC was reported in 19 (22.4%) cases. No significant difference in terms of final diagnosis was reported between the 2 scanners (*p*-value 0.7).

**Figure 1:**
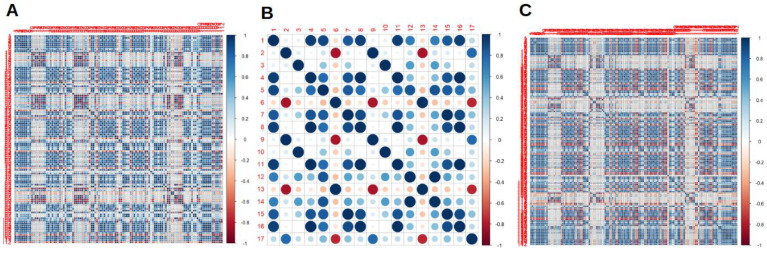
Cross correlation matrixes for radiomics features (RaF) for scanner 1 (**A**), scanner 2 (**B**) and both the scanner considered together (**C**).

RaF selection analyses and cross correlation matrixes before applying the ML models for all the scanners are presented in Figure 1. After the cross-correlation selection, scanner 2 had more removed variables, compared to scanner 1 and both the scanners considered together.

The best results of LR analysis for scanner 1, scanner 2 and both the scanners together are presented in Table 2. In this setting, for scanner 1 L_least, F_cm.2.5Dmerged.diff.entr, L_major and F_cm_2.5D.diff.entr were between the RaF with the best performances and AUCs over 0.8 were reported. On scanner 2 the best performances were obtained by F_cm.clust.shade, F_cm_merged.inv.var, F_cm.inv.var and F_cm_merged.clust.shade, with AUCs comprised between 0.6 and 0.8. When considering the combination of the two scanner together L_major, F_rlm.2.5Dmerged.sre, F_stat.entropy and F_rlm_2.5D.sre were part of the couples with the best performances, again with AUCs comprised between 0.6 and 0.8.

**Figure 2:**
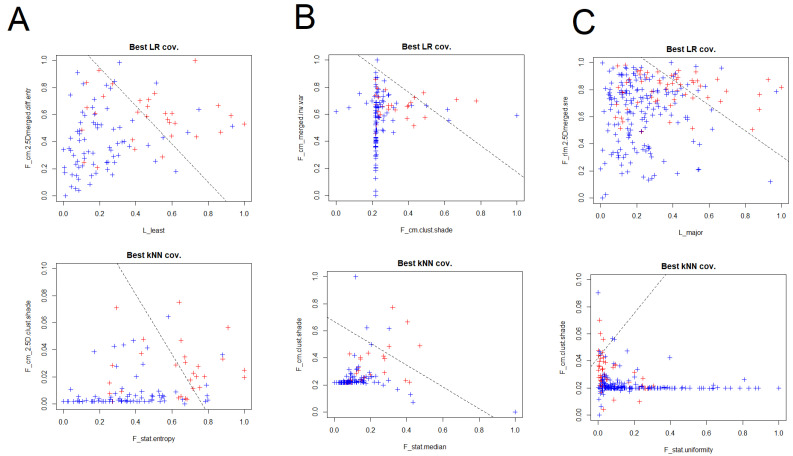
Visual representation with the couples of RaF with best performances at LR and k-nearest neighbors (kNN) analyses for scanner 1 (**A**), scanner 2 (**B**) and both the scanner considered together (**C**).

The couples of variables that presented the best performances at kNN analyses for all the scanners are presented in Table 3. For scanner 1 F_stat.entropy, F_cm_2.5D.clust.shade, F_cm.2.5Dmerged.clust.shade, F_morph.surface, F_cm_merged.clust.prom and F_cm.clust.prom had the best performances, with general AUCs above 0.8. Moreover, for scanner 2 the best performances were obtained by F_stat.median, F_cm.clust.shade, F_cm_merged.clust.shade and F_cm.joint.max, with the AUCs values that were comprised between 0.6 and 0.8. In the general analysis considering all the scanner, F_stat.uniformity, F_cm.clust.shade, F_cm_merged.clust.shade, F_rlm.lre, F_cm.2.5Dmerged.sum.entr and F_cm_2.5D.clust.shade were part of the couples of RaF with best performances, with AUCs above 0.8. In this setting, a visual representation of the best combination of RaF for kNN and LR are presented in Figure 2.

**Figure 3:**
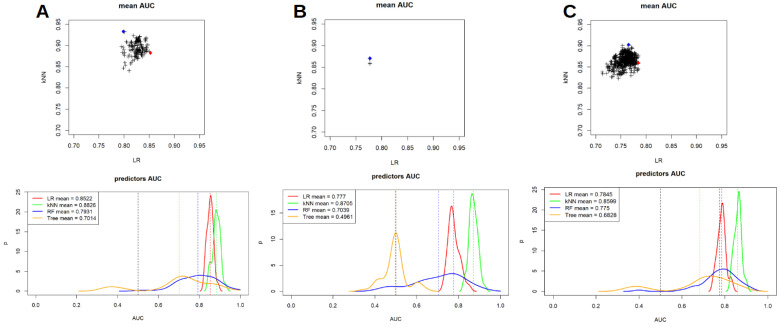
Comparison between LR, kNN, random forest and tree models performances for scanner 1 (**A**), scanner 2 (**B**) and both the scanner considered together (**C**).

Furthermore, RF and TM were also applied to our cohort and a comparison of the performances of such analyses with LR and kNN for all the PET/CT scanners are presented in Figure 3 and Table 4. In this setting, TM had the lower performances compared to other models and both TM and RF had AUC values that were lower and more heterogeneous compared to LR and kNN. In particular, for scanner 1 mean AUC for LR, kNN, RF and TM were 0.852, 0.882, 0.793 and 0.701, respectively; for scanner 2 mean AUC were 0.777, 0.870, 0.703 and 0.496, respectively, while when considering both the scanner together such values were 0.784, 0.859, 0.775 and 0.682, respectively.

## 4. Discussion

As previously underlined in the literature, the technology of the scanner used to acquire PET/CT images directly afflicts the subsequent extraction of RaF and, in this setting, the use of different tomographs in the same department is frequent in daily practice [7,17,18]. These insights suggest that different scanners can potentially have different preferred features in terms of correlations with a clinical outcome and that radiomics models coming from centers adopting different technologies should be critically considered. In our cohort, we had to deal with the presence of two different scanners and our results confirmed this point. Due to the different technologies, for example, we reported a decreasing order of AUCs values for LR trained/tested, respectively on scanner 1, both scanners and on scanner 2. As expected, the *p*-values of the features in the related models were lower for scanner 1, both scanners and scanner 2, respectively. Moreover, the selection of RaF before applying any ML models selected only a small sample of features for scanner 2 compared to scanner 1 and the analysis for both scanners. This evidence was confirmed also when considering the kNN model, with generally higher AUC values and lower *p*-values for scanner 1 and both the scanner compared to scanner 2. The higher performance of kNN can be probably due to the linear limitations of the LR modes; on the other hand, LR provides a more communicative model which can be easily plotted in a 2D space or shaped in form of normogram. Our findings are therefore in line with the concept that different PET/CT technologies can influence the contouring and/or the feature values, and therefore the performances, of RaF. In contrast with our findings, Ma et al. [20] revealed that in a large cohort of NSCLC, for most RaF the influence of different scanner on their extraction was not present. However, this is not completely unexpected: in their study they used two different tomographs with a different technological gap and different reconstructing protocols and acquisition parameters. Even if, in their case, the gap between the two scanners was not pivotal, it still remains, in general, an open challenge in this kind of analysis.

Our study was performed by comparing different ML models. In this setting, the models with best performances in the analyses for all the scanners were kNN and LR and, in general, the kNN model had better performances compared to the others. A possible reason of the highest performances of kNN can be due to its non-linearity in cutting the space. This can surely be a pros but, on the other hand, it can carry to a higher risk of overfitting. In addition, a kNN model is computational expensive (to make a prediction the DSS needs to compare the distances with all the cases in the data base) and cannot resumed in an easy-to-use graphical representation. On the other hand, LR has lower but similar performances, reduces the risk of overfitting, clearly show the role of the features in terms of *p*-value and can be easily reshaped in form of easy-to-use nomogram. In general, in choosing the best predictor, all the aforementioned points should be qualitatively considered, to reach the best trade-off for the specific needs. RF and TM had lower mean AUCs compared to the aforementioned models, and moreover such values were really heterogeneous in each analysis based on the single scanner. Furthermore, as previously mentioned, the impact of the scanner on such analyses is clearly evident and also in this scenario this evidence is confirmed: mean AUC values for scanner 2 were the lowest compared to scanner 1 and both the scanner considered together.

In general, our results underline the ability of [^18^F]FDG PET/CT RaF to discriminate between ADK and SCC in stage I and stage II NSCLC. The first study to investigate the ability of RaF in this setting was proposed by Ha et al. [23] who reported that these NSCLC entities had different tumoral heterogeneity, with 15 RaF that were able to discriminate between them and that a linear discriminant analysis with such parameters was able to clearly classify them with high performances. More recently, Kim et al. [24] reported that tumor heterogeneity of [^18^F]FDG uptake was significantly different between ADK and SCC and such parameter was able to predict recurrence of ADC but not SCC in patients who have undergone curative surgery. Orhlac et al. [22] reported the role of different resampling method on RaF in distinguish between ADC and SCC, underling that textural parameters using absolute resampling can vary in function of the cancer subtype more than in relative resampling. An interesting study by Ma et al. [20] investigated the role of texture and colour analyses in differentiating between NSCLC subtypes in a large cohort. They revealed that a combination of both methods had higher performances compared to single method in differentiating between SCC and ADK, with an AUC of 0.89. More recently, Bianconi et al. [19] reported that SCC had significantly higher degree of heterogeneity, stronger variability and lower uniformity of [^18^F]FDG uptake compared to other subtypes, while ADK had lower heterogeneity, weaker variability and higher uniformity compared to other subtypes of NSCLC. Lastly, Aydos et al. [21] revealed that kurtosis was the only RaF able to differentiate between such histological subtypes, given the fact that in SCC it was significantly lower compared to ADK. Interestingly, some RaF were also able to differentiate between moderate and poorly differentiated ADK.

Generally speaking, even with a high degree of heterogeneity in terms of number of patients, number of scanners used, methods used for RaF extraction and analyses of such parameters, a promising role for texture analysis in differentiating between NSCLC has emerged. In this setting, our study confirms such evidence, reflecting also the influence of different scanners in this context that, as mentioned, can be frequent in daily practice.

Our work is not without limitations. First of all, this is a retrospective study. Second, conventional PET/CT scanners but not last-generation tomographs are used. Moreover, even if characterized by the presence of a relatively high number of patients, in particular compared to similar works, the sample of patients included still appears sub-optimal to clearly evaluate the predictive abilities of texture analysis. The fact that the two tomographs used in our work had different reconstruction algorithm can be another confounding factor. Lastly, the problem of the reproducibility of radiomics analysis in terms of multicentric evaluation is still an open issue and further research in this field is mandatory.

## 5. Conclusions

In conclusion, our study enabled the selection of some [^18^F]FDG PET/CT RaF and ML models that are able to predict with good performances the histological subtype of NSCLC. Furthermore, evident influences of the type of PET/CT scanner on such performances were underlined.

## Figures and Tables

**Table 1:** Characteristics of the 227 patients included in the study.

Characteristic	*n* (%)
Sex	
Male	147 (64.8%)
Female	80 (35.2%)
Age (mean ± SD, range)	70 ± 8, 38–87
Histology	
Adenocarcinoma	169 (74.4%)
Squamous cell carcinoma	58 (25.6%)
Size (mean ± SD, range) (mm)	32 ± 15, 7–69
Grading *	
G1	1 (1.0%)
G2	42 (42.0%)
G3	57 (57.0%)
Lobe	
LSL	54 (23.8%)
LIL	39 (17.2%)
RSL	82 (36.1%)
ML	7 (3.1%)
RIL	45 (19.8%)
TNM stage	
T1mi	1 (0.4%)
T1a	26 (11.5%)
T1b	31 (13.7%)
T1c	11 (4.8%)
T1aN1	2 (0.9%)
T1bN1	3 (1.3%)
T1cN1	1 (0.4%)
T2a	59 (26.0%)
T2b	23 (10.1%)
T2aN1	23 (10.1%)
T2bN1	9 (4.0%)
T3	38 (16.7%)
AJCC stage	
I	
IA1	27 (11.9%)
IA2	31 (13.7%)
IA3	11 (4.8%)
IB	59 (26.0%)
II	
IIA	23 (10.1%)
IIB	76 (33.5%)
Nodal metastasis	
Yes	38 (16.7%)
No	189 (83.3%)
PET/CT scanner	
Scanner 1 (Discovery 690)	142 (62.6%)
Scanner 2 (Discovery STE)	85 (37.4%)

* Data available only for 100 patients. SD: standard deviation; mm: millimeters; LSL: left superior lobe; LIL: left inferior lobe; RSL: right superior lobe; ML: middle lobe; RIL: right inferior lobe; AJCC: American Joint Commission on Cancer; PET/CT: positron emission tomography/computed tomography.

**Table 2:** Couples of RaF with best performances at logistic regression analysis for scanner 1, scanner 2 and both scanners together.

Covariate 1	Covariate 2	Mean AUC	SD AUC	Mean *p*-Value 1	Mean *p*-Value 2
Scanner 1					
L_least	F_cm.2.5Dmerged.diff.entr	0.852	0.015	<0.001	0.020
L_least	F_cm_2.5D.diff.entr	0.850	0.017	<0.001	0.020
L_major	F_cm_2.5D.diff.entr	0.847	0.018	<0.001	<0.001
L_major	F_cm.2.5Dmerged.diff.entr	0.846	0.019	<0.001	<0.001
F_cm_2.5D.diff.entr	F_cm_2.5D.inv.diff.mom.norm	0.845	0.019	<0.001	<0.001
F_cm_2.5D.diff.entr	F_cm.2.5Dmerged.inv.diff.mom.norm	0.845	0.019	<0.001	<0.001
F_cm_2.5D.diff.entr	F_szm_2.5D.zsnu	0.845	0.018	0.012	<0.001
F_cm.2.5Dmerged.diff.entr	F_cm.2.5Dmerged.inv.diff.mom.norm	0.844	0.019	<0.001	<0.001
F_cm.diff.entr	F_cm_2.5D.inv.diff.mom.norm	0.844	0.017	<0.001	<0.001
F_cm.diff.entr	F_cm.2.5Dmerged.inv.diff.mom.norm	0.844	0.016	<0.001	<0.001
Scanner 2					
F_cm.clust.shade	F_cm_merged.inv.var	0.777	0.027	0.029	0.021
F_cm.inv.var	F_cm.clust.shade	0.777	0.028	0.019	0.030
F_cm.inv.var	F_cm_merged.clust.shade	0.777	0.028	0.019	0.030
F_cm_merged.inv.var	F_cm_merged.clust.shade	0.777	0.027	0.021	0.029
Scanner 1 + 2					
L_major	F_rlm.2.5Dmerged.sre	0.784	0.020	<0.001	<0.001
F_stat.entropy	F_rlm.2.5Dmerged.sre	0.784	0.020	<0.001	<0.001
L_least	F_rlm.2.5Dmerged.sre	0.784	0.021	<0.001	<0.001
F_stat.entropy	F_rlm_2.5D.sre	0.784	0.020	<0.001	<0.001
L_major	F_rlm_2.5D.sre	0.784	0.020	<0.001	<0.001
L_least	F_rlm_2.5D.sre	0.784	0.021	<0.001	<0.001
L_minor	F_rlm.2.5Dmerged.sre	0.782	0.020	<0.001	<0.001
L_minor	F_rlm_2.5D.sre	0.782	0.020	<0.001	<0.001
F_morph.surface	F_rlm.2.5Dmerged.sre	0.782	0.021	<0.001	<0.001
F_szm_2.5D.zsnu	F_rlm.2.5Dmerged.sre	0.782	0.020	<0.001	<0.001

AUC: area under the curve; SD: standard deviation; RaF: radiomics features.

**Table 3:** Couples of RaF with best performances at k-nearest neighbors analysis for scanner 1, scanner 2 and both scanners together.

Covariate 1	Covariate 2	Mean AUC	SD AUC	Mean *p*-Value 1	Mean *p*-Value 2
Scanner 1					
F_stat.entropy	F_cm_2.5D.clust.shade	0.938	0.012	<0.001	0.271
F_stat.entropy	F_cm.2.5Dmerged.clust.shade	0.938	0.012	<0.001	0.272
F_morph.surface	F_cm_merged.clust.prom	0.936	0.016	<0.001	0.613
F_morph.surface	F_cm.clust.prom	0.936	0.016	<0.001	0.613
F_stat.entropy	F_cm_merged.clust.prom	0.935	0.013	<0.001	0.610
F_stat.entropy	F_cm.clust.prom	0.935	0.013	<0.001	0.611
F_cm.energy	F_cm.2.5Dmerged.inv.diff.mom.norm	0.933	0.015	0.046	0.008
F_stat.entropy	F_cm_2.5D.clust.prom	0.933	0.013	<0.001	0.515
F_stat.entropy	F_cm.2.5Dmerged.clust.prom	0.933	0.013	<0.001	0.516
F_cm.energy	F_cm_2.5D.inv.diff.mom.norm	0.932	0.014	0.044	0.007
Scanner 2					
F_stat.median	F_cm.clust.shade	0.903	0.014	0.065	0.026
F_stat.median	F_cm_merged.clust.shade	0.909	0.014	0.063	0.026
F_cm.joint.max	F_cm.clust.shade	0.899	0.025	0.014	0.140
F_cm.joint.max	F_cm_merged.clust.sade	0.897	0.024	0.014	0.139
F_cm.clust.shade	F_cm_2.5D.joint.max	0.893	0.016	0.111	0.028
F_cm.2.5Dmerged.energy	F_cm.2.5Dmerged.clust.shade	0.892	0.021	0.013	0.791
F_cm_2.5D.joint.max	F_cm_merged.clust.shade	0.892	0.016	0.028	0.111
F_cm_2.5D.clust.shade	F_cm.2.5Dmerged.energy	0.892	0.022	0.790	0.013
F_cm.clust.shade	F_cm.2.5Dmerged.joint.max	0.886	0.020	0.108	0.031
F_cm_merged.clust.shade	F_cm.2.5Dmerged.joint.max	0.885	0.020	0.107	0.031
Scanner 1 + 2					
F_stat.uniformity	F_cm.clust.shade	0.912	0.011	0.008	0.110
F_stat.uniformity	F_cm_merged.clust.shade	0.911	0.011	0.008	0.111
F_rlm.lre	F_cm.2.5Dmerged.sum.entr	0.910	0.010	0.857	<0.001
F_stat.uniformity	F_cm_2.5D.clust.shade	0.907	0.014	0.008	0.158
F_stat.uniformity	F_cm.2.5Dmerged.clust.shade	0.907	0.015	0.008	0.158
F_morph.volume	F_rlm_2.5D.gl.var	0.903	0.013	0.117	0.030
F_rlm.lre	F_cm_2.5D.sum.entr	0.903	0.013	0.838	<0.001
F_morph.volume	F_rlm.2.5Dmerged.gl.var	0.903	0.014	0.117	0.031
F_stat.uniformity	F_cm.diff.avg	0.903	0.014	0.008	0.005
F_stat.uniformity	F_cm.dissimilarity	0.903	0.014	0.008	0.005

AUC: area under the curve; SD: standard deviation; RaF: radiomics features.

**Table 4:** Predictive efficiency (AUC) of ML models for scanner 1, scanner 2 and both scanners together.

ML Model	Scanner 1	Scanner 2	Scanner 1 + 2
LR	0.852	0.777	0.784
kNN	0.882	0.870	0.860
RF	0.793	0.704	0.775
DT	0.701	0.496	0.682

AUC: area under the curve; ML: machine learning; LR: Logistic Regressor; kNN: k-Nearest Neighbors; RF: Random Forest; DT: Decision Tree.

## Data Availability

Data available on request due to privacy/ethical restrictions.

## References

[1] Park S.Y., Cho A., Yu W.S., Lee C.Y., Lee J.G., Kim D.J., Chung K.Y. (2015). Prognostic value of total lesion glycolysis by ^18^F-FDG PET/CT in surgically resected stage IA non-small cell lung cancer. J. Nucl. Med..

[2] Bray F., Ferlay J., Soerjomataram I., Siegel R.L., Torre L.A., Jemal A. (2018). Global cancer statistics 2018: GLOBOCAN estimates of incidence and mortality worldwide for 36 cancers in 185 countries. CA Cancer J. Clin..

[3] Rami-Porta R., Bolejack V., Giroux D.J., Chansky K., Crowley J., Asamura H., Goldstraw P., on behalf of the International Association for the Study of Lung Cancer Staging and Prognostic Factors Committee, Advisory Board Members and Participating Institutions (2014). The IASLC lung cancer staging project: The new database to inform the eighth edition of the TNM classification of lung cancer. J. Thorac. Oncol..

[4] Duma N., Santana-Davila R., Molina J.R. (2019). Non-Small Cell Lung Cancer: Epidemiology, Screening, Diagnosis, and Treatment. Mayo Clin. Proc..

[5] Goldstraw P., Chansky K., Crowley J., Rami-Porta R., Asamura H., Eberhardt W.E., Nicholson A.G., Groome P., Mitchell A., Bolejack V. (2016). The IASLC lung cancer staging project: Proposals for revision of the TNM stage groupings in the forthcoming (eighth) edition of the TNM classification for lung cancer. J. Thorac. Oncol..

[6] Kocher F., Hilbe W., Seeber A., Pircher A., Schmid T., Greil R., Auberger J., Nevinny-Stickel M., Sterlacci W., Tzankov A. (2015). Longitudinal analysis of 2293 NSCLC patients: A comprehensive study from the TYROL registry. Lung Cancer.

[7] Gallamini A., Zwarthoed C., Borra A. (2014). Positron Emission Tomography (PET) in Oncology. Cancers.

[8] Albano D., Gatta R., Marini M., Rodella C., Camoni L., Dondi F., Giubbini R., Bertagna F. (2021). Role of ^18^F-FDG PET/CT Radiomics Features in the Differential Diagnosis of Solitary Pulmonary Nodules: Diagnostic Accuracy and Comparison between Two Different PET/CT Scanners. J. Clin. Med..

[9] Martini N., Bains M.S., Burt M.E., Zakowski M.F., McCormack P., Rusch V.W., Ginsberg R.J. (1995). Incidence and local recurrence and second primary tumors in resected stage I lung cancer. J. Thorac. Cardiovasc. Surg..

[10] Goya T., Asamura H., Yoshimura H., Kato H., Shimokata K., Tsuchiya R., Sohara Y., Miya T., Miyaoka E. (2005). Japanese Joint Committee of Lung Cancer Registry. Prognosis of 6644 resected non-small cell lung cancers in Japan: A Japanese lung cancer registry study. Lung Cancer.

[11] Harpole D.H., Herndon J.E., Young W.G., Wolfe W.G., Sabiston D.C. (1995). Stage I non-small cell lung cancer: A multivariate analysis of treatment methods and patterns of recurrence. Cancer.

[12] Dondi F., Albano D., Cerudelli E., Gazzilli M., Giubbini R., Treglia G., Bertagna F. (2020). Radiolabelled PSMA PET/CT or PET/MRI in hepatocellular carcinoma (HCC): A systematic review. Clin. Transl. Imaging.

[13] Albano D., Dondi F., Gazzilli M., Giubbini R., Bertagna F. (2021). Meta-Analysis of the Diagnostic Performance of ^18^F-FDG-PET/CT Imaging in Native Valve Endocarditis. JACC Cardiovasc. Imaging.

[14] Williams D.E., Pairolero P.C., Davis C.S., Bernatz P.E., Payne W.S., Taylor W.F., Uhlenhopp M.A., Fontana R.S. (1981). Survival of patients surgically treated for stage I lung cancer. J. Thorac. Cardiovasc. Surg..

[15] Wisnivesky J.P., Henschke C., McGinn T., Iannuzzi M.C. (2005). Prognosis of stage II non small cell lung cancer according to tumor and nodal status at diagnosis. Lung Cancer.

[16] Al-Sarraf N., Gately K., Lucey J., Aziz R., Doddakula K., Wilson L., McGovern E., Young V. (2008). Clinical implication and prognostic significance of standardised uptake value of primary non-small cell lung cancer on positron emission tomography: Analysis of 176 cases. Eur. J. Cardiothorac. Surg..

[17] Dondi F., Pasinetti N., Gatta R., Albano D., Giubbini R., Bertagna F. (2022). Comparison between Two Different Scanners for the Evaluation of the Role of ^18^F-FDG PET/CT Semiquantitative Parameters and Radiomics Features in the Prediction of Final Diagnosis of Thyroid Incidentalomas. J. Clin. Med..

[18] Sollini M., Cozzi L., Antunovic L., Chiti A., Kirienko M. (2017). PET Radiomics in NSCLC: State of the art and a proposal for harmonization of methodology. Sci. Rep..

[19] Bianconi F., Palumbo I., Fravolini M.L., Chiari R., Minestrini M., Brunese L., Palumbo B. (2019). Texture Analysis on [^18^F]FDG PET/CT in Non-Small-Cell Lung Cancer: Correlations Between PET Features, CT Features, and Histological Types. Mol. Imaging Biol..

[20] Ma Y., Feng W., Wu Z., Liu M., Zhang F., Liang Z., Cui C., Huang J., Li X., Guo X. (2018). Intra-tumoural heterogeneity characterization through texture and colour analysis for differentiation of non-small cell lung carcinoma subtypes. Phys. Med. Biol..

[21] Aydos U., Ünal E.R., Özçelik M., Akdemir D., Ekinci Ö., Taştepe A.İ., Memiş L., Atay L.Ö., Akdemir Ü.Ö. (2021). Texture features of primary tumor on ^18^F-FDG PET images in non-small cell lung cancer: The relationship between imaging and histopathological parameters. Rev. Esp. Med. Nucl. Imagen Mol..

[22] Orlhac F.M., Soussan M., Chouahnia K., Martinod E., Buvat I. (2015). 18F-FDG PET-derived textural indices reflect tissue-specific uptake pattern in non-small cell lung cancer. PLoS ONE.

[23] Ha S., Choi H., Cheon G.J., Kang K.W., Chung J.K., Kim E.E., Lee D.S. (2014). Autoclustering of Non-small Cell Lung Carcinoma Subtypes on ^18^F-FDG PET Using Texture Analysis: A Preliminary Result. Nucl. Med. Mol. Imaging.

[24] Kim D.H., Jung J.H., Son S.H., Kim C.Y., Hong C.M., Oh J.R., Jeong S.Y., Lee S.W., Lee J., Ahn B.C. (2015). Prognostic Significance of Intratumoral Metabolic Heterogeneity on 18F-FDG PET/CT in Pathological N0 Non-Small Cell Lung Cancer. Clin. Nucl. Med..

[25] van Gómez López O., García Vicente A.M., Honguero Martínez A.F., Soriano Castrejón A.M., Jiménez Londoño G.A., Udias J.M., León Atance P. (2014). Heterogeneity in [^18^F]fluorodeoxyglucose positron emission tomography/computed tomography of non-small cell lung carcinoma and its relationship to metabolic parameters and pathologic staging. Mol. Imaging.

[26] Nioche C., Orlhac F., Boughdad S., Reuzé S., Goya-Outi J., Robert C., Pellot-Barakat C., Soussan M., Frouin F., Buvat I. (2018). LIFEx: A Freeware for Radiomic Feature Calculation in Multimodality Imaging to Accelerate Advances in the Characterization of Tumor Heterogeneity. Cancer Res..

[27] Dinapoli N., Alitto A.R., Vallati M., Gatta R., Autorino R., Boldrini L., Damiani A., Valentini V. (2015). Moddicom: A complete and easily accessible library for prognostic evaluations relying on image features. Annu. Int. Conf. IEEE Eng. Med. Biol. Soc..

[28] Zwanenburg A., Vallières M., Abdalah M.A., Aerts H.J.W.L., Andrearczyk V., Apte A., Ashrafinia S., Bakas S., Beukinga R.J., Boellaard R. (2020). The Image Biomarker Standardization Initiative: Standardized Quantitative Radiomics for High-Throughput Image-based Phenotyping. Radiology.

